# Membrane filtration device for studying compression of fouling layers in membrane bioreactors

**DOI:** 10.1371/journal.pone.0181652

**Published:** 2017-07-27

**Authors:** Mads Koustrup Jørgensen, Thomas Vistisen Bugge, Poul Larsen, Per Halkjær Nielsen, Morten Lykkegaard Christensen

**Affiliations:** 1 Department of Chemistry and Bioscience, Aalborg University, Fredrik Bajers Vej 7H, Aalborg Øst, Denmark; 2 Grundfos Singapore Pte. Ltd., 25 Jalan Tukang, Singapore; 3 Dansk Miljørådgivning A/S, Fanøgade 17, Jerslev, Denmark; University of Notre Dame, UNITED STATES

## Abstract

A filtration devise was developed to assess compressibility of fouling layers in membrane bioreactors. The system consists of a flat sheet membrane with air scouring operated at constant transmembrane pressure to assess the influence of pressure on resistance of fouling layers. By fitting a mathematical model, three model parameters were obtained; a back transport parameter describing the kinetics of fouling layer formation, a specific fouling layer resistance, and a compressibility parameter. This stands out from other on-site filterability tests as model parameters to simulate filtration performance are obtained together with a characterization of compressibility. Tests on membrane bioreactor sludge showed high reproducibility. The methodology’s ability to assess compressibility was tested by filtrations of sludges from membrane bioreactors and conventional activated sludge wastewater treatment plants from three different sites. These proved that membrane bioreactor sludge showed higher compressibility than conventional activated sludge. In addition, detailed information on the underlying mechanisms of the difference in fouling propensity were obtained, as conventional activated sludge showed slower fouling formation, lower specific resistance and lower compressibility of fouling layers, which is explained by a higher degree of flocculation.

## Introduction

Membrane bioreactors (MBR) are now widely applied in municipal and industrial wastewater treatment, but membrane fouling remains one of the major obstacles in reducing operational costs [1]. Numerous studies have proposed different approaches to mitigate fouling, e.g. by optimized operational modes [2], adding antifouling agents [3], using antifouling membranes [4], and using different configurations of the system [1]. Also, identification of critical potential foulants and operational conditions leading to membrane fouling has been studied intensively, e.g. with multiple regression analysis, to understand the fouling phenomena and with this knowledge reduce the problem [5]. However, several parameters influence fouling propensity, e.g. particle size distribution, suspended solids levels and presence of filamentous bacteria [6]. It is therefore difficult to apply such specific parameters for fouling control. Alternatively, the fouling propensity of individual sludge samples can be determined from filtration experiments and data from such experiments can be used to optimize MBR operations. Accordingly, a range of methods for on-site filterability assessment has been developed, focusing on the MBR process, including the Berlin Filtration Method (BFM) [7], Delft Filtration Characterization method (DFCm) [8], and the MBR VITO Fouling Measurement (MBR-VFM) [9]. The MBR-VFM uses a tubular membrane to filtrate MBR sludge with a constant flux and assess filterability through evolution of transmembrane pressure (TMP) and filtration resistance. The protocol includes relaxation periods to assess fouling reversibility [10]. DFCm has been used in a range of studies to assess fouling propensity with an external sidestream membrane, operated in constant flux mode. Fouling propensity is expressed through the fouling resistance evolved by producing 20 L permeate per m^-2^ membrane and with a compressibility coefficient showing insignificant compressibility in the high shear crossflow filtrations [11]. The BFM setup consists of a test cell with a flat sheet membrane that can be immersed directly into the bioreactor and the fouling propensity is calculated based on a flux-step protocol with relaxation steps, which enables determinations of critical flux and fouling reversibility.

Fouling models can be a useful tool to understand and interpret the fouling behavior in MBR systems [12]. Various fouling models for MBR systems have been proposed, ranging from mechanistic models based on Darcy's law to data-driven approaches such as artificial neural networks and models based on principal component analysis [12]. Drews et al. [13] presents an approach based on the resistance-in-series model dividing fouling resistance into resistances from different fouling mechanisms (cake layer, pore blocking, gel layer, etc.), combined with individual models for the resistances from the cake layer and pore blocking. With this, Drews and colleagues can determine the dominating fouling mechanism at different stages of the filtration process. Bugge et al. [14] introduced cake compressibility in this filtration model. Hence, the resistance of the fouling layer is pressure dependent with higher pressures elevating resistance, which also has been underlined by Naessens et al. [12]. The effect of compressibility has successfully been included into a model to simulate data obtained from fouling experiments, and from key fitting parameters the fouling behavior in lab-scale MBR systems is interpreted [14–16].

In this study, a new fully automated, on-site filtration test system (AaFPA, Aalborg Filtration Property Analyzer) has been built to characterize fouling layer compressibility and its effect on fouling layer resistance during short term filtration. Hence, focus was on the short term fouling mechanisms, excluding biofilm growth. The filtration system was a flat sheet system with aerated membranes. To control and study compression effects, the filtrations were performed at fixed TMP and elevated in steps (TMP step experiments) with intermittent relaxation steps to assess the compressibility of fouling. Key fouling parameters describing kinetics of fouling layer formation and fouling layer pressure dependency have been obtained by fitting the modified model to filtration data, assuming that cake formation and compression are the dominating mechanisms of fouling.

## Materials and methods

### Design of filtration system

**Fig 1:**
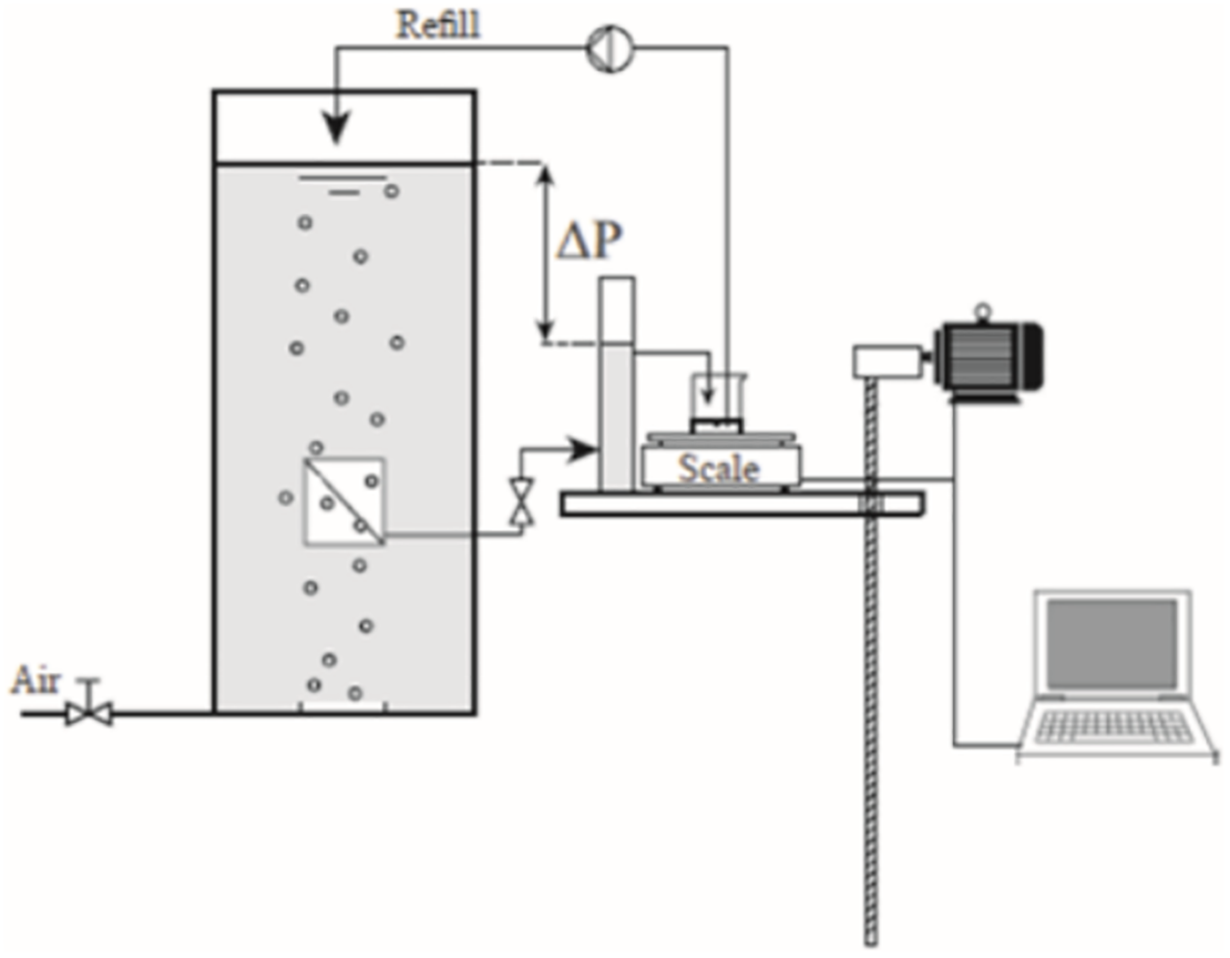
AaFPA filtration property analyzer for quantification of sludge fouling propensity.

The principle of the AaFPA step filtration system is shown in Fig 1.

The AaFPA device consists of four main parts: a reactor, a flat sheet membrane, a TMP control unit, and software for collecting flux data and management of the filtration test cycles with filtration at different TMPs and relaxation.

The reactor was prepared from two transparent acrylic plates (1300 × 300 × 10 mm). The reactor space between the plates was prepared by cutting a 20 mm thick “U”-shaped acrylic plastic frame, which was glued on to one of the 10 mm thick acrylic plates. The second 10 mm thick plate was mounted with nuts and bolts with a packing between the frame and the second plate. In this way, the reactor could be completely disassembled and cleaned. The reactor volume was 7.8 L and 5.0 L of sludge sample was used for each filtration. In the bottom of the 20 mm frame, four holes (0.5 mm diameter each) were made for air scouring, and an airflow meter with control valve was mounted to enable accurate control over air scouring. The flow was set to 3529 L air×h^-1^×m^-2^ membrane, corresponding to a gas flow velocity of approximately 5.6×10^−3^ m×s^-1^, which is in the same range as that applied to the full-scale plants by Alfa Laval.

An immersed flat sheet membrane was installed. The flat sheet membrane was placed 200 mm above the bottom of the reactor. Two flat metal frames with a hollow plastic spacer (70×60×5 mm) were designed, which make it possible to test flexible flat sheet membranes such as the MFP2 PVDF membrane from Alfa Laval used in this study. The metal frames are “U”-shaped with holes for bolts and nuts to assemble and tighten the flat sheet membrane, with an area of 0.0084 m^2^. The inner size of the “U” fitted the size of the hollow plastic spacer, and a piece of membrane was cut so that it could be folded around the plastic spacer, and the metal frames could be mounted from each side with a small pipe connected to the hollow plastic spacer. The membrane was placed in the reactor with the folded side of the membrane, i.e. the open side of the “U” in the metal frames facing down towards the air scouring. In this manner, hydraulic effects from the metal frame were minimized, and the membrane was directly exposed to the air scouring. The pipe from the membrane was connected to an external permeate pipe via a magnetic valve (Buschjost 8253000.8001, Buschjost GmbH). Relaxation was started by shutting the valve, which prevented permeate from passing through the membrane.

The TMP control unit consisted of a permeate pipe with a fixed overflow leading permeate to a scale that measures permeate flux by weight over time. The TMP was adjusted by the level difference between the permeate collector and sludge level in the reactor, *ΔP*, hence TMP = *ΔP*. The scales were prepared from a 0–500 g load sensor (SEN128A3B, Seeedstudio), mounted with an instrumentation amplifier (INA 125, Farnell.com). The load data was collected with an analog data collection module (Measurement computing-USB-1208LS, Measurement Computing), which was connected to Matlab 11. The TMP control unit was equipped with a membrane pump (Evaki EVN-b11VC), which empties the scales back into the reactor each time the load reaches 40 ml to reduce effect on TMP. The scales and the permeate pipe were mounted on a spindle actuator with a geared DC-motor to move fixed permeate overflow in the vertical direction relatively to top level of sludge in the membrane tank. Movement and thereby adjustment of TMP was performed during relaxation periods.

All valves and pumps were controlled via a relay board from Phidgets (PhidgetInterfaceKit 0/0/4, Phidgets Inc., CA) with four relays for individual control of devices running on 250VAC/10A or 100VDC/5A. A Matlab program was developed for integrated data collection and control of TMP and filtration/relaxation cycles. All parameters are fully changeable, and, once initiated, the filtration experiments run completely automatically and flux/TMP data is stored for further processing/modelling.

### MBR sludge samples for filtration experiments

Sludge samples were collected from different wastewater treatment facilities to assess the ability of the AaFPA setup to reproduce data and assess the difference in filtration propensity between sludge samples. The samples came from three different wastewater treatment facilities; Aalborg Vest Wastewater Treatment Plant (WWTP), Bjerringbro WWTP and Lundtofte WWTP, all with CAS and MBR systems running in parallel. It took up to 24 hours before the samples arrived at the laboratory; filtration and analyses were started immediately upon arrival. MBR sludge from Aalborg Vest WWTP was analyzed by triplication, whereas sludge samples from remaining sites were only filtrated once. The long time could influence on the filtration properties of the sludge samples, which would also be reflected by the analysis of sludge characteristics.

The pilot MBR at Aalborg Vest WWTP was provided by Alfa Laval A/S and used to treat pre-clarified raw wastewater, treated in a conventional nutrient removing WWTP. The MBR was operated with a membrane module stack (40 m^2^) of “hollow sheet” PVDF membranes (MFP2, Alfa Laval A/S, Denmark). The MBR system at Bjerringbro WWTP consisted of a Grundfos BioBooster system with sidestream tubes with rotating ceramic membrane discs to create high shear for filtration of activated sludge. The system was operated with biological nutrient removal. The hydraulic conditions in the Grundfos BioBooster MBR were not comparable to the AaFPA filtration device, but the sludge was expected to have high fouling potential due to shear deflocculation and served as a sample for comparison of sludge samples’ fouling propensity. The MBR system at Lundtofte WWTP consisted of four lines, each consisting of six Zeeweed ® 500 membrane modules for filtration of activated sludge treated with denitrification.

Sludge samples were analyzed by measuring total suspended solids concentration (TSS, equal to concentration of sludge (*C*_b_, kg×m^-3)^), pH, conductivity, particle size distribution, capillary suction time (CST, s), residual turbidity, total, dissolved and extracted extracellular polymeric substances (EPS). TSS was measured in accordance with standards given by APHA et al. [17]. pH was measured with a pH meter (PHM 290, Radiometer Analytical, France) equipped with a pH electrode (Blueline, Schott Instruments, Germany) and conductivity with a conductivity meter (CDM 210, Radiometer Analytical, France). The size distribution of sludge flocs and particles was analyzed by laser diffraction (LS 13 320, Beckman Coulter, CA, USA) and CST was measured with a CST meter (Model 304B CST, Triton Electronics Ltd., UK) equipped with standard filter paper. The residual turbidity of sludge samples was determined by centrifugation of sludge samples for 2 min at 3000 rpm (854 x g) (Model 1–6, Sigma Laboratory Centrifuges, Germany) and measuring the turbidity of the supernatant as the absorbance at 650 nm wavelength (Model Helios Epsilon, Thermo Spectronic, USA).

### Filtration protocol

The filterability test procedure was inspired by Bugge et al. [14] and consisted of 10 steps (*n = 1–10*) of varying TMP in the order 1000 Pa, 1500 Pa, 1950 Pa, 2450 Pa, 2950 Pa, 3425 Pa, 4000 Pa, 4500 Pa, 5000 Pa. Each step lasted 60 min and 60 min relaxation phases were used between each step in order to fully restore permeability. This procedure was carried out three times over four days on Aalborg Vest MBR sludge to determine the reproducibility of the method, and applied one time for each of the other sludge samples. An alternative procedure was carried out, where each step was reduced to 15 min of filtration which resulted in a total filtration time of 4 h and 45 min to reduce experiment time and the risk for changes in sludge characteristics.

Prior to filtration tests, the membrane resistance was determined by measuring permeate flux at pressures in the 1000–7000 Pa range and from these data calculating membrane resistance:

$$R_{m}=\frac{TMP}{J\mu }$$ (1)

where *μ* is the dynamic viscosity of permeate (Pa×s), TMP is the transmembrane pressure (Pa), and *J* is the permeate flux (m×s^-1^). The membrane resistance was determined to be 1.49×10^11^ m^-1^ ± 0.06×10^11^ m^-1^. After tests, the membrane unit was cleaned with NaOCl (soaked in 1000 ppm solution for at least 2 hours) and then stored in demineralized water.

### Mathematical assessment of filterability parameters

The fouling model proposed in Bugge et al. [14] was applied to interpret filtration data. The model was derived by assuming that cake formation and compression is the main fouling mechanism in short term MBR filtration experiments and that pore blocking, adsorption, and gel layer formation are negligible. For a full description of the modeling procedure, see [14]. The flux, *J* (m×s^-1^) was calculated from Eq (2).

$$J=\frac{TMP}{\mu (R_{m}+R_{c})}$$ (2)

*R*_c_ is the cake layer’s resistance (m^-1^) and is the product of the specific cake mass, *ω* (kg×m^-2^), and the average specific cake resistance, *α* (m×kg^-1^). Several studies have shown that there is a power law relationship between specific resistance and pressure. One often used equation is Eq (3A) [18]

$$\alpha =\alpha_{0}{\left(1+\frac{TMP}{P_{a}}\right)}^{n}$$ (3A)

where *α*_0_ (m×kg^-1^) is the specific resistance of an uncompressed cake, and *n* and *P*_a_ (Pa) are compressibility constants. For cakes formed by wastewater sludge, there is a linear dependency between pressure and resistance due to its extreme compressibility i.e. *n* is equal to 1, [19,20]. Thus, Eq 3A can be reduced to Eq 3B

$$\alpha =\alpha_{0}+\frac{\alpha_{0}}{P_{a}}TMP$$ (3B)

In Eq 3B, *P*_a_ (Pa) is the pressure where the resistance increases to 2*α*_0_, and the cake compressibility can be described from the ratio *α*_0_*/P*_a_, i.e. the slope of the *α*-TMP plot. It follows that a low value of *P*_*a*_ is associated with a high elevation in specific resistance, thus, high compressibility.

The development in amount of cake is modeled by solving the following mass balance numerically [14]:

$$\frac{d\omega }{dt}=\left(J-J_{LIM}(1-e^{-\omega /\omega_{crit}})\right)C_{b}$$ (4)

*ω*_crit_ is the critical specific mass of cake and *J*_LIM_ is the limiting flux (m×s^-1^). During TMP step filtrations the flux declines towards a steady state which increases with the level of TMP, until the limiting flux, *J*_LIM_, is reached [15]. The limiting flux is the flux at which the convective transport of foulants towards the membrane equals the back transport induced by shear, i.e. it is a measure of the degree of back transport. As the difference between flux and limiting flux determines the rate of cake buildup, a high limiting flux corresponds to low fouling rate.

The flux is simulated for the TMP step experiments with Eq 2 and by numerical solution of Eq 4. Sludge concentration, *C*_b_, membrane resistance, *R*_m_, permeate viscosity, μ, and TMP at the given step have known values, whereas the values of *J*_LIM_, *α*_0_ and *P*_a_ are unknown. It follows from Eq 3 that the same value for *α* can be obtained by varying *α*_0_ and *P*_a_. Therefore, it has been decided to adjust *J*_LIM_ and *α(*TMP*)*, instead of fitting *J*_LIM_, *α*_0_ and *P*_a_. This is beneficial, as it is easier to obtain stable and reliable fitting parameters having two instead of three adjustable parameters. The modelled flux is fitted to the measured flux by minimizing the RMSE by using the SOLVER function in Microsoft Excel. This procedure has been repeated for all TMP steps by adjusting *J*_LIM_ and *α(TMP)* and initially assuming *ω*(t = 0) to equal zero and *ω*_crit_ to 0.055 kg×m^-2^ [14]. As the initial resistance in some TMP steps deviates from zero, the initial amount of cake (*ω*(t = 0)) and the critical amount of cake, *ω*_crit_ were adjusted. By adjusting *ω*(t = 0) for each TMP step it is taken into account that irreversible fouling occurs and that not all fouling can be described from the reversible fouling model.

## Results and discussion

### TMP step filtration data

**Fig 2:**
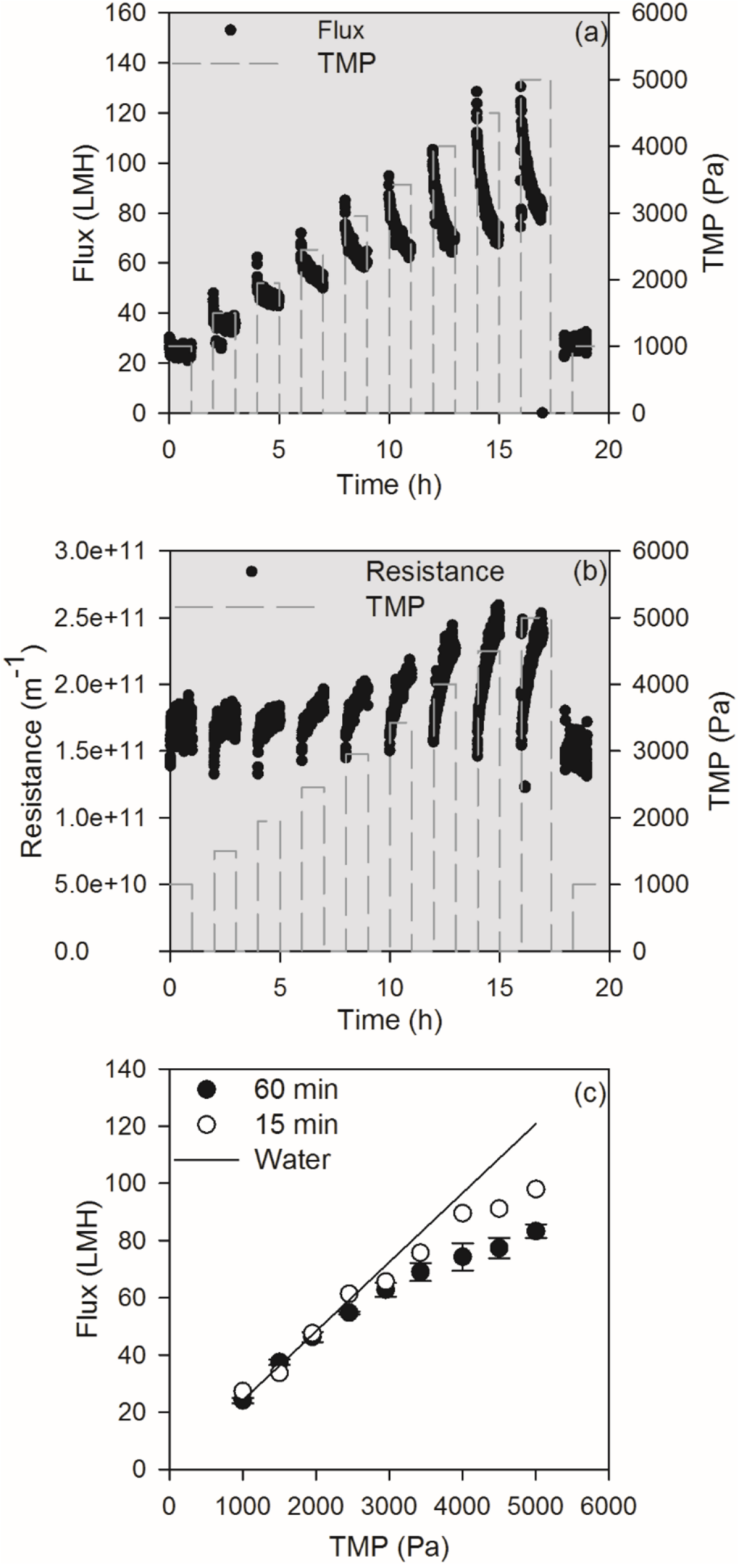
The development of flux (a) and resistance (b) over time measured for 60 min TMP step experiments with MBR sludge from Aalborg Vest wastewater treatment plant, and end fluxes vs. TMP from 60 min and 15 min filtration experiments of (c). The error bars represent standard deviation from the triplicated measurements.

Flux and resistance for TMP step filtrations (19 hour tests) of sludge from Aalborg Vest MBR pilot plant are shown in Fig 2. Flux data are shown in the units of L×m^-2^×h^-1^ (LMH). The fluxes obtained in filtration experiments with Aalborg Vest sludge were significantly higher than the fluxes measured in the actual Aalborg Vest pilot MBR (10–15 L×m^-2^×h^-1^). Part of the difference is due to irreversible fouling accumulating in the pilot MBR, as the membranes in the pilot MBR were fouled over a longer period increasing the resistance of the membrane [21].

At low TMP, the fluxes were low, and there was only a slight decline in flux over time. As TMP was elevated, the initial flux increased, but it also resulted in a more significant decline in flux over time. Hence, for TMP above 3000 Pa, the flux converged towards the same steady state flux. The flux continued declining after 60 min filtration; thus, not achieving a steady state. This was also observed for the 15 min TMP step experiment (data not shown). Plotting the end fluxes against TMP for the 60 min filtrations (Fig 2C) shows increasing end flux with increasing TMP, with linearity at TMP < 2000 Pa. i.e. the critical flux occurred at TMP < 2000 Pa. At TMP 4000–5000 Pa, the flux of the 15 min TMP step experiments was more than 10 L×m^-2^×h^-1^ higher than the fluxes found for the 60 min filtrations. There was no clear difference between fluxes at TMP below 4000 Pa.

The resistance during filtration of Aalborg Vest MBR sludge (Fig 2B) was calculated from Eq (2). The resistance increased, starting from the clean membrane resistance (~1.49×10^11^ m^-1^), indicating fouling of the membrane. At higher pressures, the elevation in resistance during the one hour filtration steps was higher. The graph also shows that the resistance was reduced to a level close to the membrane resistance after relaxation, i.e. relaxation was able to reestablish the flux by removing fouling. The resistance was 1.69×10^11^ ± 0.08×10^11^ m^-1^ during the first part of the filtration (TMP = 1000 Pa), while it was lower during the last part (1.53×10^11^ ± 0.06×10^11^ m^-1^). This tendency was also observed during the other TMP step experiments, and shows that it is fair to assume that only reversible fouling is formed during the experiment.

### Fitting fouling model to filtration data

**Fig 3:**
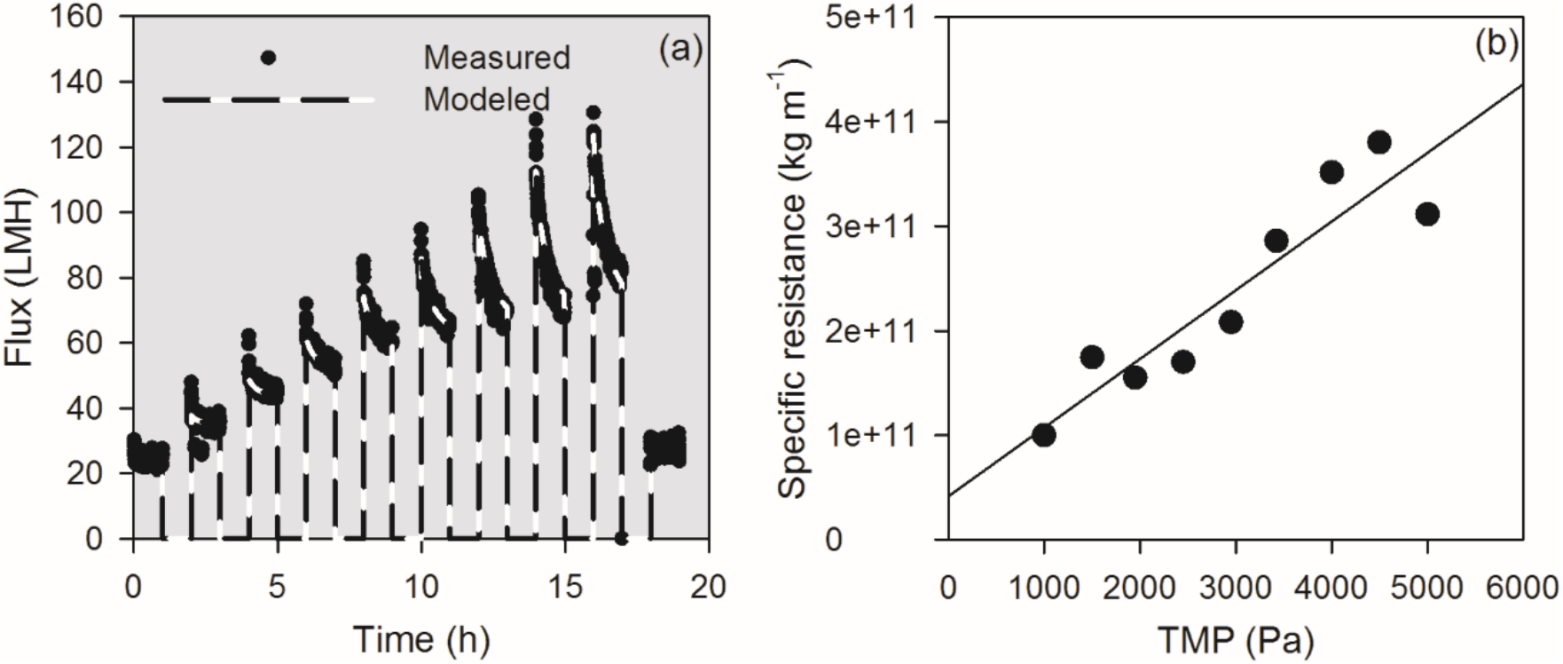
Plot of measured and modeled flux vs. time for Aalborg Vest MBR 60 min filtration experiment (a) and specific resistances proposed by the solver function in Microsoft Excel in the fitting procedure (b).

For all TMP step filtrations, the flux was modeled by using the procedure outlined in Fig 3. The modeled data was fitted to experimental fluxes by adjusting *J*_LIM_ and *α*(TMP). By doing this, one *J*_LIM_ value and one *α* were found for each TMP. In Fig 3A, the measured and modeled flux is plotted for a filtration with 60 min TMP steps. Fig 3B shows the specific resistances found during the TMP step procedure.

Fig 3A shows the modeled flux after adjusting limiting flux and specific resistance. After each relaxation period, *ω* = 0; hence *ω*(*t* = 0) was set to zero for all TMP steps in filtrations of Aalborg Vest MBR sludge. Accordingly, the relaxation was an effective method for fouling removal, and irreversible fouling was negligible throughout the filtrations, and the numerical model was able to describe the development in flux.

For all filtration experiments, the specific cake resistances increased with TMP, exemplified by data from 60 min shown in Fig 3B. A linear regression of the plot shows that *α*(*TMP*) = 6.58 × 10^7^ × *TMP* + 4.19 × 10^10^. It follows from Eq 3 that the intercept with the y-axis equals α_0_, and the slope of the regression equals α_0_/*P*_*a*_, from which *P*_*a*_ was determined. During the second fitting step *J*_LIM_, *α*_0_, *P*_a_, and *ω*_crit_ were fitted, using α_0_ and *P*_a_ determined from the linear regression as initial estimates. The fitted parameters are listed in Table 1 together with the characteristics of sludge samples.

**Table 1:** Sludge characteristics and filtration properties.

	Filtration	15 min	60 min (1)	60 min (2)	60 min (3)	Average (60 min)
Sludge Characteristics	TSS (g×L^-1^)	7.78 ±0.14	8.09 ±0.60	7.77 ±0.19	7.67 ±0.13	7.83 ±0.33
	pH	7.18	7.22	7.26	7.20	7.23 ±0.05
	Conductivity (μS×cm^-1^)	1175	1115	1104	1273	1189 ±120
	Mean floc size (μm)	34.1	31.0	31.2	29.6	30.6 ±0.9
	Residual turbidity (-)	0.085 ±0.014	0.111 ±0.077	0.077 ±0.022	0.092 ±0.022	0.093 ±0.022
	CST (sec)	47.0 ± 6.0	46.3 ±2.3	52.6 ±1.4	49.4 ±3.9	49.4 ±3.6
Filtration properties	*α*_0_ (m×kg^-1^)	3.42×10^11^	1.71×10^11^	1.12×10^11^	1.32×10^11^	1.38 ±0.30×10^11^
	*P*_a_ (Pa)	7685	3282	2251	3054	2863 ±542
	*α*_0_ / *P*_a_ (m×kg^-1^×Pa^-1^)	4.45×10^7^	5.21×10^7^	4.97×10^7^	4.33×10^7^	4.84 ±0.45×10^7^
	*J*_*LIM*_ (L×m^-2^×h^-1^)	74.0	68.0	71.1	71.2	70.1 ±1.8
	*ω*_*crit*_ (kg×m^-2^)	0.090	0.090	0.093	0.089	0.091 ±0.002
	RMSE of model fit	7.94	7.19	8.32	8.76	8.09

Sludge characteristics and filtration properties assessed for MBR sludge from Aalborg Vest Wastewater Treatment Plant.

Table 1 shows that the TSS concentration was stable for the MBR sludge from Aalborg Vest WWTP during the sampling period of five days and measured to be 7.83 ± 0.33 g×L^-1^. pH was measured to be 7.23 ± 0.05, conductivity was 1189 ±120 μS×cm^-1^, and mean floc size was 30.6 ± 0.9 μm. For the sludge sample for the first 60 min TMP step, the residual turbidity was higher than for the following samples, but with high standard deviation. Apart from this the characteristics of the sludge samples did not vary significantly.

By averaging the values determined from the 60 min TMP step (Aalborg Vest MBR sludge), *J*_LIM_ was found to be 70 ±1.8 L×m^-2^×h^-1^, α_0_ to be 1.4×10^11^ ±0.30×10^11^ m×kg^-1^, and compressibility parameter α_0_/*P*_*a*_ was 4.84×10^7^ ±0.45×10^7^ m×kg^-1^×Pa^-1^. Hence, the filtration method shows a high degree of reproducibility (see Fig 2C) as well as the model fitting procedure. For comparison, the parameters obtained for the 15 min TMP step filtrations (Aalborg Vest MBR sludge) were slightly to some extent different from the parameters for 60 min TMP steps; *J*_LIM_ was slightly higher at 74 L×m^-2^×h^-1^, α_0_ was determined to be 3.63×10^11^ m×kg^-1^, and the compressibility, *P*_*a*_ was 7685 Pa, and α_0_/*P*_*a*_, was 4.45×10^7^ m×kg^-1^×Pa^-1^. Especially, there is a more significant difference in specific resistance and *P*_a_ in-between the 5h and 18h filtration procedure. However, the determination of these two parameters (individually) is difficult as they depend on each other. Hence, it could be that the values of specific resistance and *P*_a_ are the same for the two samples, but because of the fitting process, they are different. What is important is that the ratio between these, α_0_/*P*_a_ is the same. This ratio corresponds to the compressibility of the cake, and that is the same for the 5h and 18h operations. Hence, *J*_LIM_ and compressibility are determined in the same order of magnitude by the 5h filtration procedure as for the 18h filtration procedure. It follows that 15 min filtration steps are sufficient for determination of compressibility parameters, and it is unnecessary to apply 60 min filtration steps, which takes longer time. In addition, the correlance between 15 min and 60 min filtration data implies that the longer filtration time did not influence on sludge filtration characteristics.

### Relation to sludge characteristics

To test the methodology’s ability to characterize compressibility of sludge with different characteristics and filtration properties, sludge samples from 6 different MBR and CAS systems were characterized with the method. The physico-chemical characteristics (Table 2) represents the sludge samples at arrival after transport from the full-scale plants. Minor changes compared to the original sample may have taken place due to transportation.

**Table 2:** Characteristics and filtration properties of different sludges.

	Plant	Aalborg Vest MBR	Aalborg Vest CAS	Bjerringbro MBR	Bjerringbro CAS	Lundtofte MBR	Lundtofte CAS
Sludge characteristics	TSS (g×L^-1^)	7.83 ±0.33	2.70	11.3	5.02	9.8	7.37
	pH	7.23 ±0.05	6.80	6.78	7.29	7.28	7.22
	Conductivity (μS×cm^-1^)	1189 ±120	692	713	697	1111	1013
	CST/TSS (s×L×g^-1^)	6.30±0.5	4.20	8.11	2.46	3.62	2.86
	Residual Turbidity (-)	0.093 ±0.022	0.019	0.190	0.006	0.032	0.026
	Mean floc size (μm)	30.6 ±0.9	70.6	62.5	100.2	43.5	46.9
Filtration properties	J_LIM_ (L×m^-2^×h^-1^)	70.1 ±1.8	82.3	22.0	66.0	36.0	48.0
	*ω*_crit_ (kg×m^-2^)	0.09	0.10	0.05	0.05	0.18	0.10
	*α*_0_ (m kg^-1^)	0.138±0.030×10^12^	1.62×10^12^	4.25×10^12^	0.91×10^12^	1.04×10^12^	1.99×10^12^
	*P*_a_ (Pa)	2863±542	10800	14616	45428	8922	35300
	Compressibility, *α*_0_/*P*_a_ (m kg^-1^ Pa^-1^)	0.48 ±0.05×10^8^	1.50×10^8^	2.91×10^8^	0.20×10^8^	1.17×10^8^	0.56×10^8^

Characteristics and filtration properties of sludge sampled from CAS and MBR systems at Aalborg Vest, Bjerringbro and Lundtofte wastewater treatment facilities.

The table shows that the MBR sludges had significantly higher TSS, residual turbidity, and CST/TSS and lower floc size than CAS sludge samples, which indicates a lower degree of flocculation. The high residual turbidity and low floc size of the MBR samples compared to CAS samples show that the degree of flocculation was lower. Especially Bjerringbro MBR sludge showed low degree of flocculation, probably due to shear of the sludge by disc rotation in the BioBooster system. Additionally, the filtration properties found by model fit to filtration data show lower back transport (*J*_LIM_, i.e. faster flux decline according to Eq 4) and higher compressibility, *α*_0_/*P*_a_, during filtration of MBR sludge compared to CAS sludge. For the Aalborg West and Lundtofte WWTP samples, the specific resistance (α_0_) was lower during filtration of MBR sludge than CAS sludge. These observations are in correlation with literature, where a lower degree of flocculation of MBR samples gives lower filtration properties due to shear and higher TSS [22–24].

By correlation analysis on the small set of data, a Pearson correlation coefficient for linear change of *J*_LIM_ vs TSS is found to *r*_P_ = -0.91, which shows that the limiting flux is significantly decreasing with TSS. This is explained by the decrease in concentration gradient between bulk and membrane surface lowering the back transport from shear induced diffusion. Hence, TSS influences only back transport and not filtration properties in general, as filtration properties is also governed by specific resistance and compressibility, which are not governed by TSS. This can explain the lack of correlation between filtration flux and TSS reported in previous studies [25]. Therefore, the methodology presented in this study gives more detailed information on fouling propensity of sludge, as the fouling layer morphology (specific resistance and compressibility) are assessed to give more detailed understanding on how different parameters influence fouling.

## Conclusions

A new device (AaFPA) and method for assessment of fouling layer compressibility has been developed by combining automated TMP step filtrations and numerical modelling of fouling. The method characterizes fouling layer compressibility by steps of filtrations at constant TMP, which through a simple model fit gives the main output parameters *J*_LIM_, *α*_0_ and compressibility; *α*_0_/*P*_a_. The system proved to be a flexible and reliable set-up, applying overflow as a controlling factor for TMP and provided accurate and simple TMP control, which, together with the spindle actuator, was very flexible and easy to control.

The methodology was tested to quantify compressibility of MBR sludges with different characteristics. This on-site filtration method can provide useful information about the fouling propensity of sludge under varying conditions; comparable to the information provided by existing methods, but through the combination with the fouling model, empirical parameter outputs can be determined as well. This gives more detailed information on the morphology and compressibility of a fouling layer which opens for a more detailed understanding of the dependency of fouling.

## Supporting information

S1 Data Data from filtration experiments. (XLSX)
